# Editorial: “The cognitive, emotional and neural correlates of creativity”

**DOI:** 10.3389/fnhum.2015.00275

**Published:** 2015-05-19

**Authors:** Matthijs Baas, Bernard A. Nijstad, Carsten K. W. De Dreu

**Affiliations:** ^1^Department of Psychology, University of AmsterdamAmsterdam, Netherlands; ^2^Department of Human Resource Management and Organizational Behavior, University of GroningenGroningen, Netherlands

**Keywords:** creativity, divergent thinking, incubation, motivation, emotion, EEG, neuroscience

Creativity is at the roots of extraordinary achievements in the arts and sciences, and enables individuals and their groups to adapt flexibly to changing circumstances, to manage complex social relations, and to survive and prosper through social, technological, and medical innovations. The ability to generate novel and potentially useful ideas and problem solutions (viz., creativity) is a key driver of human evolution, and among the most valued and sought after competencies in contemporary societies. Because creativity provides fitness functionality in both ancestral and contemporary societies, it stands to reason that (i) the human brain evolved to sustain and promote creative thinking and we should therefore be able to identify, (ii) the brain circuitries and neurohormonal modulators of the human capacity for creativity, and (iii) the core cognitive, motivational, and emotional processes underlying creative thought.

In support of these propositions, in the past decade, creativity researchers have made great headway in identifying the neural, cognitive, motivational, and emotional correlates of creativity (e.g., Baas et al., 2008; Dietrich and Kanso, 2010; Nijstad et al., 2010; De Dreu et al., 2014). This Research Topic offers a collection of empirical work, and review and opinion papers about these and other stimulating endeavors.

## Cognitive correlates of creativity

Research has shown that creative outcomes are a function of multiple cognitive processes, including divergent and flexible thinking, the use of flat and broad (as opposed to steep and narrow) associative hierarchies, convergent and persistent thinking, and incubation-driven processes (Sio and Ormerod, 2009; Nijstad et al., 2010; Baas et al., 2011).

In this Research Topic, Kenett and colleagues re-examined the classic proposition of Mednick (1962) that creative individuals are characterized by flat associational hierarchies. Using novel computational network paradigms, they revealed that the semantic memory network of low creative people seems to be more rigid, compared to the network of highly creative persons. Ritter and Dijksterhuis reviewed evidence for the intriguing possibility that creative discoveries oftentimes result from a period during which one refrains from task-related conscious thought (i.e., incubation). These authors explored possible causes of incubation effects and argue that during incubation periods unconscious processes contribute to creativity. Colzato and colleagues examined whether convergent and divergent thinking are differentially affected by acute moderate and intense physical exercise in athletes and non-athletes. Finally, Stevenson and colleagues researched whether creativity could be improved by practicing divergent thinking. Participants indeed improved in creative ideation and cognitive flexibility, with adolescents often benefitting more from training than adults.

## Emotional and motivational correlates of creativity

Past work shows that creativity and divergent thinking are triggered by appetitive cues, such as love, performing approach behavior, and mating cues (Friedman and Förster, 2010) and positive emotions, such as happiness and joy (Baas et al., 2008). Other work revealed that aversive cues and negative emotions may reduce divergent and flexible thinking (Baas et al., 2008; Byron and Khazanchi, 2011), but may nevertheless lead to enhanced creativity under the right circumstances (De Dreu et al., 2008; Baas et al., 2011; Roskes et al., 2012). In this Research Topic, Icekson and colleagues highlight the role of optimism as a potential remedy for the creativity undermining effects of avoidance motivation, due to its beneficial impact on cognitive (e.g., threat appraisals), affective (e.g., anxiety), and volitional processes (e.g., low intrinsic motivation). Oleynick, Thrash and colleagues took the formidable challenge to define and measure inspiration, a motivational state that compels individuals to bring ideas into fruition. They challenge the well-known observation by Edison that creativity is 1% inspiration and 99% perspiration and argue that both play important—but different—roles in creativity.

## Neural correlates of creativity

Exciting research has identified the (interplay among) brain regions associated with creative ideation and insight (Kounios and Beeman, 2009; Jung et al., 2010), the neurohormonal modulators, such as dopamine and oxytocin (Chermahini and Hommel, 2010; De Dreu et al., 2014), the genetic components (Reuter et al., 2006; Simonton, 2008), and important methodological problems associated with the neuroscientific study of creativity (Dietrich and Kanso, 2010).

In this Research Topic, Mok addressed the inconsistent results regarding the neural signatures of creativity, suggesting that creative cognition likely emerges from an *optimal* balance between PFC mediated controlled processing and spontaneous processing that is mediated by the default-mode network. Abraham makes a case for studying the neural correlates of distinct cognitive processes underlying creativity, to uncover the information processing brain mechanisms by which creativity occurs. Schwab and colleagues took a different approach and focused on time-related changes of EEG alpha activity patterns during creative ideation. Among other things, they discovered that the production of more original ideas was accompanied by increasing hemispheric asymmetry (more alpha in the right than left hemisphere) with increasing duration of the idea generation period. Vartanian and colleagues nicely integrated findings from sleep research with research on PFC-mediated divergent thinking. Exploring the impact of a single night of sleep deprivation on idea generation (i.e., fluency) and PFC function during divergent thinking, these authors discovered that cognitive effectiveness and fluency were impaired following sleep deprivation.

### Conflict of interest statement

The authors declare that the research was conducted in the absence of any commercial or financial relationships that could be construed as a potential conflict of interest.
